# Allogeneic Hematopoietic Stem Cell Transplantation for PEX1-Related Zellweger Spectrum Disorder: A Case Report and Literature Review

**DOI:** 10.3389/fped.2021.672187

**Published:** 2021-08-25

**Authors:** Kai Chen, Na Zhang, Jing-Bo Shao, Hong Li, Jie Li, Jia-Ming Xi, Wu-Hen Xu, Hui Jiang

**Affiliations:** ^1^Department of Hematology and Oncology, Shanghai Children's Hospital, Shanghai Jiaotong University, Shanghai, China; ^2^Department of Neurology, Shanghai Children's Hospital, Shanghai Jiaotong University, Shanghai, China; ^3^Central Laboratory, Shanghai Children's Hospital, Shanghai Jiaotong University, Shanghai, China

**Keywords:** zellweger spectrum disorder, infantile refsum disease, pex1, hematopoietic stem cell transplantation, curative effect

## Abstract

Zellweger spectrum disorder (ZSD) is a heterogeneous group of autosomal recessive disorders characterized by a defect in peroxisome formation and attributable to mutations in the *PEX* gene family. Patients with ZSD have profound neurologic impairments, including seizures, severe retardation, and dysmorphic features, and poor prognosis. Currently, there is no specific, effective treatment. Here, we investigated the effects of allogeneic hematopoietic stem cell transplantation (allo-HSCT) on *PEX1*-related ZSD. The suspected clinical proband was first diagnosed at the Department of Neurology of our hospital. The proband died soon after diagnosis, and his family was studied. We found that a brother had the same genetic alterations, and he was diagnosed with Infantile Refsum disease (IRD) as the mildest form of ZSD. We implemented treatment with allo-HSCT, at the request of the child's parents. After transplantation, we observed significant improvements in the clinical manifestations, very-long-chain fatty acids, and brain MRI. The patient has recovered well and not showed any abnormal clinical manifestations after 2 years of follow-up. We have achieved satisfactory short-term results in the treatment of ZSD-IRD with allo-HSCT. Long-term follow-up and observation will be performed to determine the long-term prognosis.

## Introduction

Zellweger spectrum disorders (ZSD) is defined by a continuum of three phenotypes and the biochemical and molecular bases of these disorders have been fully determined and include severe (Zellweger syndrome) and milder phenotypes [neonatal adrenoleukodystrophy and infantile Refsum disease (IRD)] (1). Severe ZSD manifestations include neurological impairments, stunting, and multiple congenital abnormalities involving the brain, bone, liver, eyes, kidneys, and endocrine glands (1–3). Infants with severe ZSD are significantly impaired and typically die during the first year of life, usually having made no developmental progress. Intermediate/milder ZSD have no congenital malformations, but rather progressive peroxisome dysfunction variably manifests with sensory loss and neurologic involvement (ataxia, polyneuropathy, and leukodystrophy). While hypotonia and developmental delays are typical, intellect can be normal in patients with intermediate/milder ZSD (1). Due to the extreme variability in the disease manifestations, its diagnosis and medical management present enormous challenges (1–4). This study mainly involved a pair of brothers with PEX1-related ZSD. They were diagnosed with ZSD-IRD with clinical manifestations and examination. Here, we have summarized the relevant results.

## Clinical Report

**Figure 1:**
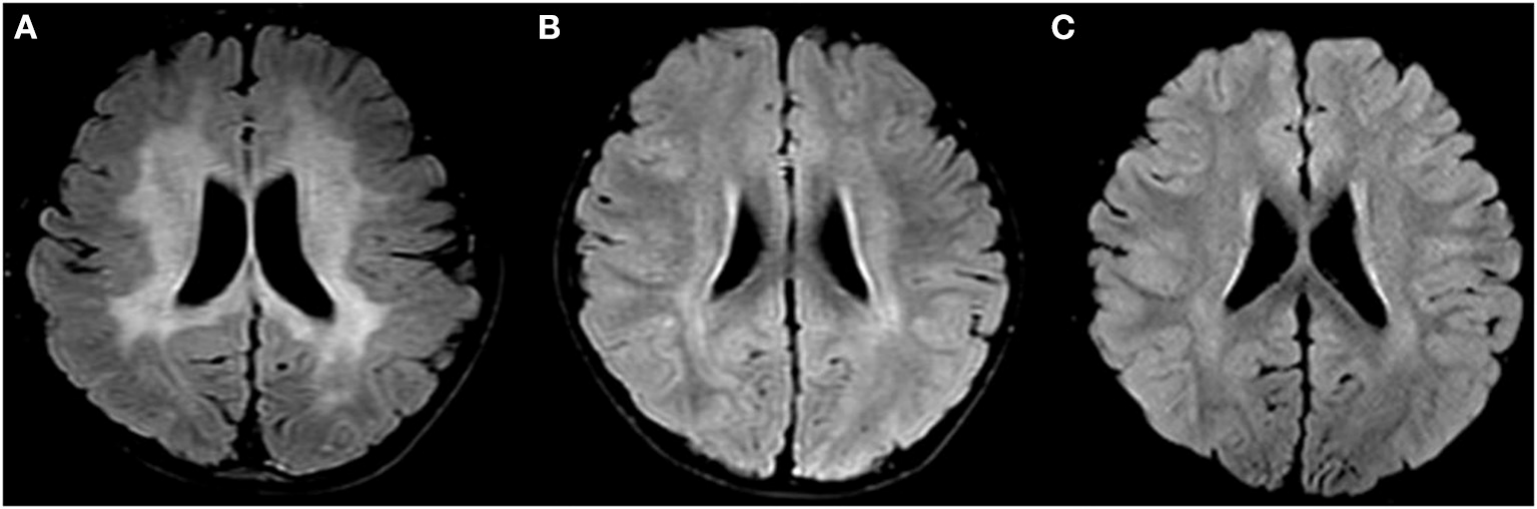
Changes in brain MRIs associated with Zellweger syndrome. **(A)** Brain MRI of patient 1, A T2-weighted FLAIR image shows high signal intensity (PHILIPS MRI). **(B)** Brain MRI of patient 2 before transplantation. A T2-weighted TIRM shows high signal intensity (SIEMENS MRI). **(C)** Brain MRI of patient 2 after transplantation. A T2-TIRM shows high signal intensity (SIEMENS MRI). FLAIR, fluid-attenuated inversion recovery; TIRM, turbo inversion recovery magnitude.

**Patient 1:** The first case was a four-year-old boy. His parents reported that he could not speak until two years of age. At the age of 3, he was diagnosed with hearing loss. At the age of 4, he was diagnosed with congenital deafness, and a hearing aid was installed. Later, he gradually exhibited an unstable gait, susceptibility to falling, and extreme fear of heights. A brain MRI showed extensive, symmetrical, flaky, abnormal signals in bilateral paraventricular white matter, the semioval center, bilateral basal ganglia, the brainstem, and the corpus callosum (Figure 1A). Moreover, we detected elevated plasma VLCFAs (C24:0, C26:0, C24:0/C22:0, and C26:0/C22:0 were obviously higher). In an exome analysis, we have found compound heterozygous mutations in *PEX1* exon 6: c.1246_1247 delGA (p.D416) of the patient's father and exon 19: c.2966T>C (p.I989T) of the patient's mother. Our final diagnosis was ZSD. The child quickly developed epilepsy, vision loss, and dysphagia. The patient survived for only five months after the diagnosis.

**Figure 2:**
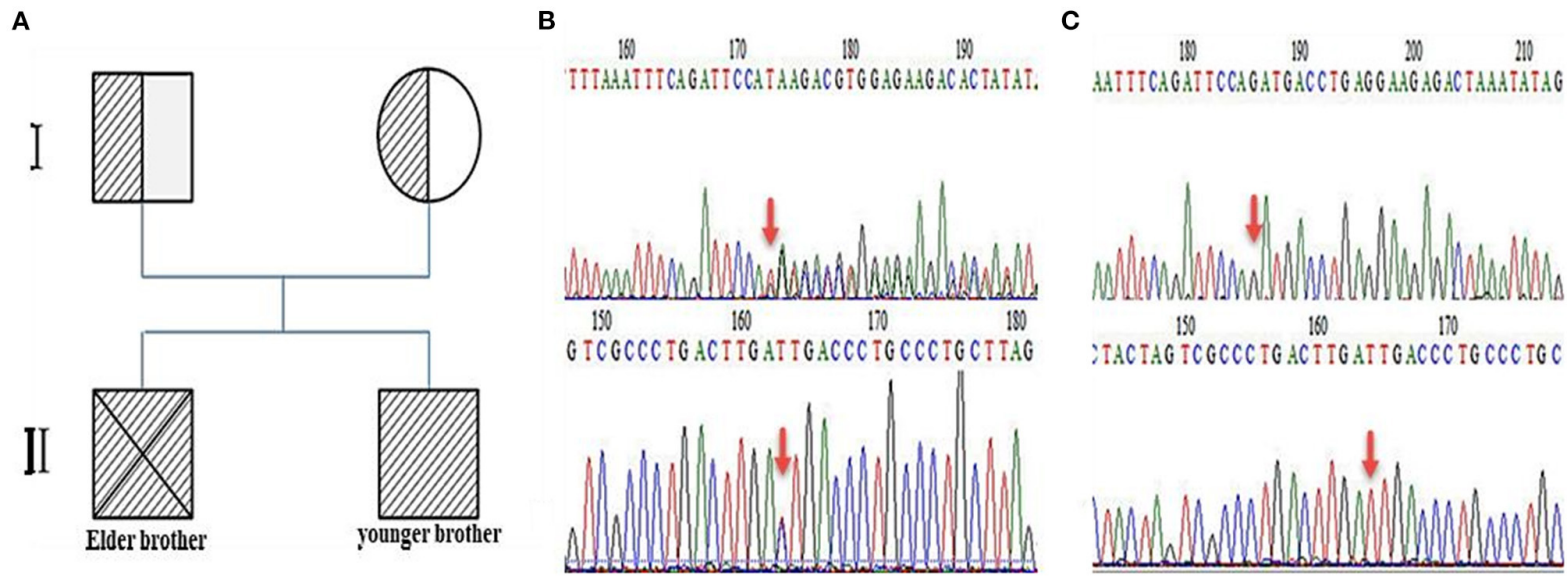
Analysis of ZS inheritance. **(A)** The family tree shows that the parents are gene carriers, the elder brother died, and the younger brother survived. **(B)** DNA sequence analyses show two mutation sites (red arrows) before transplantation, in (top) exon6: c.1246_1247 delGA (p.D416) and (bottom) exon19: c.2966T > C (p.I989T). **(C)** After transplantation, no pathogenic gene mutation was detected in (top) exon 6 or (bottom) exon 19.

**Patient 2**: The second patient was a 3-year-old boy and the younger brother of the index patient. He was admitted to our hospital on February 3rd, 2019. We found the same genetic abnormalities in the two brothers (Figure 2). A slight hearing loss, unclear expression, cryptorchidism, and elevated plasma VLCFAs (C24:0, C26:0, C24:0/C22:0, and C26:0/C22:0) were found in patient 2. A brain MRI showed patchy, abnormal signal intensities in the bilateral paraventricular white matter (Figure 1B). The diagnosis of IRD [the onset of clinical manifestations like hearing loss, loss of appetite, slow growth and development, and leukodystrophy was made at 3 years old (3)] was confirmed.

## Treatment Options and Results

There are reports about the treatment of ZSD using hepatocyte transplantation or orthotopic liver transplantation (5). These therapy modalities can improve the clinical manifestations but are not curative treatment. Hepatocyte transplantation technology is not well developed in China, and its indications are relatively limited. Orthotopic liver transplantation was not carried out because the parents refused this treatment option. Allogeneic hematopoietic stem cell transplantation (allo-HSCT) is an effective method to treat X-linked adrenoleukodystrophy (X-ALD) in childhood (6), but there is no report about HSCT in ZSD patients. Due to the rapid death of the proband brother, the young brother was considered at high risk of the same prognosis eventually. After several rounds of discussion, the MDT team reached the consensus that HSCT might be beneficial to a subgroup of patients with ZSD. Treatment protocol and approval from the ethics committee of Shanghai Children's Hospital were obtained. The parents were informed about the HSCT scheme and provided informed consent for treatment was provided.

**Figure 3:**
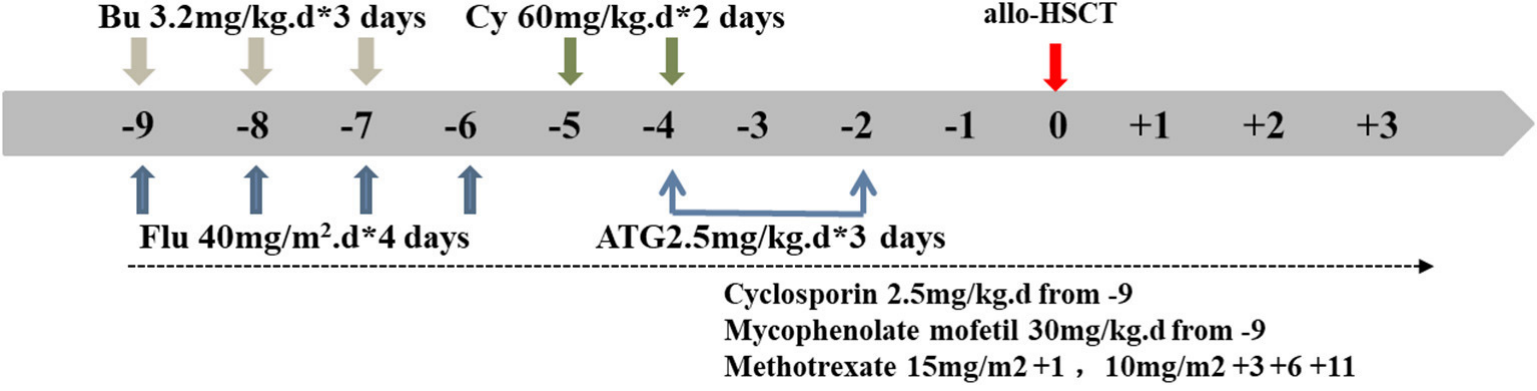
Conditioning regimen schedule. Days (d) are numbered with respect to the day of transplantation (day 0). Bu, busulfan; Flu, fludarabine; Cy, cyclophosphamide; ATG, antithymocyte globulin; allo HSCT, allogeneic hematopoietic stem cell transplant.

An unrelated matching donor (27 year old, male, HLA 10/10, consistent blood group) was found for this child in the China Marrow Donor Program. For the allo-HSCT procedure, we formulated a reduced-toxicity conditioning regimen based on busulfan (BU, Otsuka, Japan), fludarabine (Flu, Baxter, Germany), cyclophosphamide (CY, Baxter, Germany), and anti-thymocyte globulin (ATG, Sanofi, France). These drugs were administered at the following dosages (Figure 3; the day of HSCT = day 0): BU: 3.2 mg/kg intravenously (i.v.), in divided doses, daily for 3 days (total dose of 9.6 mg/kg) on days −9 to −7; Flu: 40 mg/m^2^, once daily i.v. for 4 consecutive days, on days −9 to −6; CY: 60 mg/kg, once daily i.v. for 2 consecutive days, on days −5 and −4; and ATG: 2.5 mg/kg, once daily i.v for 3 consecutive days, on days −4 to −2. The patient received cyclosporine (CsA), mycophenolate mofetil (MMF), and short-term methotrexate (MTX) as prophylaxis for acute graft-vs.-host disease. The CsA dosage was 2.5 mg/kg/day i.v., in two doses daily, from day −9; after that, the patient received oral CsA. MMF was administered orally twice daily, at 30 mg/kg/day from day −9 to day +30. MTX was administered at 15 mg/m^2^ i.v., on day +1, and at 10 mg/m^2^, on days +3, +6, and +11. The trough CsA concentration was monitored with a fluorescence polarization immunoassay. The target trough CsA blood concentration was 150-200 ng/ml until 6 months after HSCT. Thereafter, the CsA dose was gradually tapered over the following 2–3 months, until complete CsA withdrawal was achieved (Figure 3).

The patient received 21.27 × 10^8^/kg nucleated cells and 9.56 × 10^6^/kg CD34^+^ cells isolated from the peripheral blood on April 22th 2019. Engraftment was observed, based on an absolute neutrophil count of >1.0 × 10^9^/L on day +12, platelet implantation on day +16, and red blood cell implantation on day +19. A blood chimerism analysis showed more than 95% of donor cells on day +14. A DNA sequence analysis (first-generation sequencing method) showed that the *PEX1* mutations were abolished, and no abnormality was detected on day +30. Relevant tests were conducted on days +90, +180, and +360 after transplantation and the results were all negative (Figure 2C). Moreover, the plasma VLCFA levels were restored to their normal reference values from day +90 after transplantation, and the regular detection was carried out continuously (Table 1). A cranial MRI showed that the abnormal signal shadows in the bilateral paraventricular white matter area had improved (Figure 1C). At that time, a clinical examination showed no deterioration in the patient's hearing. He could communicate with his parents and express his ideas simply. Then he was discharged on May 14th, 2019. The patient was regularly followed on an outpatient basis every month for liver and kidney function tests, VLCFA examinations, and chimerism tests. Brain MRI and hearing tests were repeated every 3 months. The last follow-up date was on April 28th, 2021, 2 years post-transplantation. The patient recovered well, with no abnormal manifestations or transplant-related complications. His undergo regular observations and follow-up examinations have been continuing.

**Table 1:** Changes of plasma VLCFAs before and after hematopoietic stem cell transplantation.

**VLCFAs**	**Reference Value**	**Pre-transplant**	**Post-transplant +30d**	**Post-transplant +90d**	**Post-transplant +180d**	**Post-transplant +270d**	**Post-transplant +360d**
C22:0(mg/ml)	0.00–0.02	0.03–0.04 ↑	0.01	0.01	0.02	0.02	0.02
C24:0(mg/ml)	0.00–0.065	0.2–0.35 ↑	0.07 ↑	0.05	0.06	0.05	0.07 ↑
C26:0(mg/ml)	0.00–0.07	0.16–0.29 ↑	0.05	0.06	0.07	0.08 ↑	0.07
C24:0/C22:0	0-6.0	6.7–8.75 ↑	7 ↑	5	3	2.5	3.5
C26:0/C22:0	0–6.75	5.3–7.25 ↑	5	6	3.5	4	3.5

*VLCFAs, Very long chain fatty acids; C22:0 docosanoic acid; C24:0 tetracosenoic acid; C26:0 hexacosanoic acid. ↑represents higher than the reference value*.

## Discussion

ZSD arises from a deficiency in many peroxisome enzymes, which can lead to the accumulation of VLCFAs and deficiencies in acetals and phospholipids (1, 6, 7). The accumulations of VLCFAs can lead to lipotoxicity (1, 6, 7). Patients with ZSD exhibit delayed mental development, marked hypotonia, feeding difficulties, and respiratory impairments (1–3). Often, patients die in the early stages of life. ZSD diagnosis requires fibroblast enzyme analysis to evaluate peroxisome function or detect PEX gene mutation (1, 7), but only a few professional laboratories carry out these analyses, and we have not been able to check fibroblast enzyme further. For patients with suspected ZSD, based on clinical manifestations, a plasma VLCFA analysis can provide preliminary screening for ZSD. The most common VLCFA elevations in ZSD are observed in C26:0 and the C26:0/C22:0 and C24:0/C22:0 ratios. The plasma VLCFA analyses showed results consistent with previous studies (8, 9). As noted by the literature, the clinical course of ZSD is very variable among the patients (3). The reason of younger brother had a milder condition than his brother might be younger age and the tissue damage was not developed.

The clinical manifestations of ZSD are considered to be variable among patients (1–4). In this report, the proband exhibited deafness, unstable gait, epilepsy, vision loss, and dysphagia. His younger brother had mild disease and showed a slight hearing loss, unclear expression, and cryptorchidism, varied abnormalities on brain MRI. The phenotypes described here are globally consistent with other published cases (10–13).

ZSD treatment often requires multidisciplinary participation. Due to the lack of effective treatments, research has focused on optimizing patient quality of life. we have reported allo-HSCT for treating PEX1-related ZSD firstly. We have found that allo-HSCT achieved good effectiveness in the presented case during the short-term follow-up. In the treatment of X-ALD allo-HSCT reported 20 years ago, and has also been confirmed that it could have good long-term efficacy. At present, allo-HSCT is the an alternative therapy to prevent the progression of neurometabolic diseases. Some reports show that allo-HSCT treatment in the early stage of the disease can obtain positive results, and in patients with asymptomatic can obtain better survival rate (14, 15). Within symptomatic patients who have a high burden of cerebral white-matter disease at diagnosis, the outcome of transplantation is often unfavorable. Good survival outcomes were observed in patients who received allo-HSCT for the pre-symptomatic disease that has been detected by means of early findings on imaging studies (16). Compared with X-ALD, we have found no reports describing HSCT in ZSD patients. Due to the early death of the proband, it was considered that the younger brother was at high risk of facing the same outcome. Therefore, HSCT was suggested and accepted by the family. By far, the treatment was successful, possibly due to the following reasons. Firstly, the presented ZSD case was mild. In addition, allo-HSCT was successful, without any complications, and effectively lowered the VLCFAs level as well as alleviating the other symptoms. Furthermore, treatment was performed before disease progression. The metabolism of VLCFAs relies on peroxidase, but patients with ZSD cannot metabolize VLCFA normally due to PEX gene mutation-caused peroxidase dysfunction. After bone marrow transplantation, the PEX gene of the bone marrow cells was normal, which played a compensatory role in VLCFA metabolism. Moreover, the presented case was mild ZSD, so the VLCFA could have been restored to the normal level, and the patient has good prognosis. The long-term benefits of allo-HSCT in X-ALD are thought to be mediated by donor-derived replacement of myeloid derived cells, possibly including microglial cells (17). ZSD patients may also obtain such results through transplantation. This report cannot be generalized to all patients. HSCT could be beneficial for a subgroup of patients within the ZSD spectrum, but we need to determine how to predict which patients will progress and need intervention. Side and fatal effects of HSCT in severely affected patients should be kept in mind. The benefits of transplantation may be offset after complications.

In conclusion, allo-HSCT might be a potential treatment strategy for patients with ZSD. Long-term follow-up and further clinical trials are required to determine the feasibility of this treatment for children with PEX1-related ZSD.

## Data Availability Statement

The original contributions presented in the study are included in the article/supplementary material, further inquiries can be directed to the corresponding author/s.

## Ethics Statement

The studies involving human participants were reviewed and approved by Ethics Committee of Shanghai Children's Hospital. Written informed consent to participate in this study was provided by the participants' legal guardian/next of kin. Written informed consent was obtained from the participants' legal guardian/next of kin for the publication of this case report.

## Author Contributions

KC participated in the entire management and treatment for the case, reviewed the relevant literature on the topic, and drafted and revised the manuscript. HJ directed the entire treatment process and reviewed and revised the manuscript. All authors contributed to the article and approved the submitted version.

## Conflict of Interest

The authors declare that the research was conducted in the absence of any commercial or financial relationships that could be construed as a potential conflict of interest.

## Publisher's Note

All claims expressed in this article are solely those of the authors and do not necessarily represent those of their affiliated organizations, or those of the publisher, the editors and the reviewers. Any product that may be evaluated in this article, or claim that may be made by its manufacturer, is not guaranteed or endorsed by the publisher.
